# Suppressive Modulation of the Chick Forebrain Network for Imprinting by Thyroid Hormone: An *in Vitro* Study

**DOI:** 10.3389/fphys.2022.881947

**Published:** 2022-04-20

**Authors:** Yuriko Saheki, Naoya Aoki, Koichi J. Homma, Toshiya Matsushima

**Affiliations:** ^1^ Department of Biology, Faculty of Science, Hokkaido University, Sapporo, Japan; ^2^ Department of Molecular Biology, Faculty of Pharmaceutical Sciences, Teikyo University, Tokyo, Japan

**Keywords:** imprinting, thyroid hormone, sensitive period, NMDA receptor, GABA-A receptor

## Abstract

The thyroid hormone 3,5,3′-triiodothyronine (T_3_) is considered to act acutely in the chick forebrain because focal infusion of T_3_ to the intermediate medial mesopallium (IMM) causes 4 to 6-day-old hatchlings to become imprintable approximately 30 min after the infusion. To understand the mechanism of this acute T_3_ action, we examined synaptic responses of IMM neurons in slice preparations *in vitro*. Extracellular field potential responses to local electrical stimulation were pharmacologically dissociated to synaptic components mediated by AMPA and NMDA receptors, as well as GABA-A and -B receptors. Bath-applied T_3_ (20–40 μM) enhanced the positive peak amplitude of the field potential, which represented the GABA-A component. Bicuculline induced spontaneous epileptic bursts by NMDA receptor activation, and subsequent application of T_3_ suppressed the bursting frequency. Pretreatment of slices with T_3_ failed to influence the synaptic potentiation caused by tetanic stimulation. Intracellular whole-cell recording using a patch electrode confirmed the T_3_ actions on the GABA-A and NMDA components. T_3_ enhanced the GABA-A response and suppressed the NMDA plateau potential without changes in the resting membrane potential or the threshold of action potentials. Contrary to our initial expectation, T_3_ suppressed the synaptic drives of IMM neurons, and did not influence activity-dependent synaptic potentiation. Imprinting-associated T_3_ influx may act as an acute suppressor of the IMM network.

## 1 Introduction

Since being coined by Lorenz (1937), imprinting has amassed a long history of ethological and neuroscientific investigation as a unique form of behavioral development toward formation of social attachment in precocial animals such as domestic chicks and ducklings (Bolhuis et al., 1985; Bolhoius 1999). Beside the cellular and molecular bases of imprinting memory formation (Horn, 1998, Horn, 2004; Horn et al., 2001), considerable efforts have addressed the sensitive (or critical) period, one of the core characteristics of imprinting (Hess, 1958, Hess, 1959; Bateson, 1978). However, the mechanisms underlying this aspect of imprinting remained vague until recently.

Thyroid hormone (3,5,3′-triiodothyronine, T_3_) is the critical molecule determining the sensitive period (Yamaguchi et al., 2012). Imprinting enhances expression of the converting enzyme (type2 iodothyronine deiodinase, Dio2) in the vascular endothelial cells of the dorsal pallium (including the intermediate medial mesopallium, IMM), leading to a rapid influx of T_3_ to the neuronal network involved in imprinting. Two distinct modes of T_3_ action occur, an acute and a chronic effect. Usually, 4 to 6-day-old hatchlings cannot be imprinted, but injection of exogenous T_3_ (either systemically or focally into the IMM that is responsible for imprinting) makes them imprintable approximately 30 min after the injection; T_3_ acutely re-opens the previously-closed sensitive period. Systemic T_3_ application also increases the imprinting score in 1-day-old chicks, indicating that thyroid hormone is functional at hatching or earlier. Furthermore, imprinting on day 1, or a single injection of exogenous T_3_, makes the treated chicks imprintable to an object of novel color at 4–6-days post-hatching, when the sensitive period is normally closed; T_3_ elongates the sensitive period so that the imprinted chicks remain imprintable.

Recently, Batista et al. (2018) revealed that the mechanistic target of rapamycin complex 1 (mTORC1) is critically involved in the acute effect of thyroid hormone; its blockade by rapamycin injection impaired imprinting, and its activation re-opened the sensitive period at 4-days post-hatching, similar to thyroxine injection. Furthermore, we have found that development of GABA receptor-mediated transmission in the IMM is also critical in the control of the sensitive period (Aoki et al., 2018); systemic intravenous injection of GABA-B agonist (baclofen) made 4-day-old hatchlings imprintable, whereas its selective blockade impaired imprinting in 1-day-old hatchlings. Thyroid hormone can act on the mTORC1 kinase cascade and GABA receptors to affect behavior.

Despite these clear-cut pharmacological findings, the assumed biochemical cascades have not been causally linked to the network properties of the IMM. It is important to determine how thyroid hormone affects in the IMM neurons and synapses, and how these cellular/synaptic events change the neural network dynamics. Furthermore, which of these changes lead to the behavioral expression of social attachment in hatchlings needs to be determined.

In addition to detailed Golgi cytoarchitecture (Tömböl et al., 1988), ample histochemical and connectivity data are available for the IMM (Bradley et al., 1985; Csillag, 1999). A neuro-physiological study reported activity-dependent synaptic potentiation in the IMM (Bradley et al., 1991), although the synaptic transmission responsible was not specified. We (Yanagihara et al., 1998) then showed that a DNQX-sensitive excitatory component (namely AMPA receptor mediated) is potentiated by locally applied tetanic electrical stimulation, but the plastic change was not linked to immediate early gene (*c-fos*) expression, which was clearly associated with the imprinting score at the individual level (McCabe and Horn, 1994). Since the single-unit characterization of visual objects in the IMM (Horn et al., 2001), little progress has been made and our understanding of imprinting mechanisms remains elusive at the neuron level.

In this study, inspired by the recent findings concerning thyroid hormone, we investigated the neuronal/synaptic target of the hormonal action using IMM slice preparation *in vitro*. If T_3_ activated the IMM network, exogenous bath application of T_3_ should enhance the neuronal excitability, excitatory synaptic drive or facilitate synaptic potentiation. Alternatively, T_3_ might not modulate IMM excitability, but might act via other aspects of the neural network. As reported for cultured mammalian hippocampal neurons (Losi et al., 2008; Puia and Losi 2011), T_3_ can suppress the synaptic transmission mediated by GABA and glutamate receptors. This also seems to be the case in chicks, as we show here.

## 2 Materials and Methods

### 2.1 Slice Preparation

Domestic chicks of a white leghorn strain (called “Julia” by the local name of hatchery; 2–5 days post-hatching, mostly 3 days old) were used. Fertilized eggs were purchased from a local supplier (Sankyo Labo Service Co., Sapporo, Japan; Iwamura Poultry Co., Yubari, Japan; Nippon White Farm Co., Shiretoko Farm, Abashiri, Japan) and incubated in the laboratory. To avoid possible complication due to uncontrolled learning experiences, hatchlings were individually housed in cages placed in a dark incubator at 37°C until used in experiments. Chicks were decapitated under a deep anesthesia by i. m. injection of 0.8 ml ketamine-xylazine cocktail; a 1:1 mixture of 10 mg/ml ketamine hydrochloride (Daiichi-Sankyo Propharma Co., Tokyo, Japan) and 2 mg/ml xylazine (Sigma-Aldrich Co., St Louis, Missouri, United States ). The left hemisphere of telencephalon was dissected out, and quickly immersed in ice-chilled Krebs solution composed of (in mM): NaCl, 113; NaHCO_3_, 25.0; KCl, 4.7; KH_2_PO_4_, 1.2; CaCl_2_, 2.5; MgSO_4_, 1.2, glucose, 11.1; pH was adjusted to 7.2–7.4 by bubbling the solution with 95% O_2_—5% CO_2_. Using a vibrating microslicer (DTK-1000, Dosaka EM Co., Kyoto, Japan), two to three 400 μm-thick coronal slices were cut from the brain and stored for recovery (>1 h) in an interface-type slice chamber at room temperature (ca. 26–28 °C) supplied with a continuous flow of humid O_2_/CO_2_ gas. Thereafter, slices were placed in a submersion-type recording chamber (ca. 2 ml in volume), which was continuously perfused with Krebs solution held at 30 °C at a flow rate of 1.5 ml/min. The following drugs were applied to the perfusing solution: 3,5,3′-triiodo-l-thyronine (T_3_, Sigma Co.); 6,7-dinitroquinoxaline-2,3-dion (DNQX, Tocris Bioscience Co.); 1(S),9(R)-(-)-bicuculline methiodide (bicuculline, Sigma Co.); D (-)-2-amino-5-phosphonovaleric acid (D-AP5, Wako Co.) [3-[[(3,4-dichlorophenyl)methyl]amino]propyl](diethoxymethyl)-phosphinic acid (CGP52432, Tocris Bioscience Co.). Concentration of receptor blockers in the perfusing bath solution was fixed at: DNQX (10 μM), bicuculline (10 μM), D-AP5 (50 μM), and CGP52432 (1 μM). When Ca^2+^ was removed, no divalent cation was substituted.

### 2.2 Electrical Stimulation and Recording of Extracellular Field Potential

Electrical stimulation was delivered via a concentric metal electrode (o.d. = 300 μm; UB-9007, Unique Medical Co., Tokyo Japan) placed on the slice surface. The center (or sleeve) of the electrode was connected to the cathode (or anode) of the isolating unit (SS-203 J) and pulse generator (SEN-3301, Nihon Koden Co., Tokyo, Japan) in the constant current mode. Field potential was recorded using a pair of Krebs-solution-filled glass micropipettes (o.d. = 1.2 mm, i. d. = 0.69 mm, borosilicate glass; #BF120-69-10, Sutter Instruments Co., Novato, California, United States ) with fine tips made by pulling and cutting (o.d. = ca. 50 μm). One pipette (plus pole) was placed in the slice tissue, and another (minus pole) was submerged in the perfusing Krebs solution as the reference electrode. These pipettes were connected through inserted silver wires to a differential amplifier (DAM-50, World Precision Instruments Inc. Sarasota, Florida, United States ) set at frequency range of 1 to 1,000 Hz and gain of x1,000. The amplified signals were stored in a computer via an A/D interface and sampling software (Power 1401 and Signal ver 5, Cambridge Electronic Design Ltd. Cambridge, United Kingdom) after digitizing at 5 kHz.

### 2.3 Whole-Cell Intracellular Recording by Nystatin-Perforation Patch Recording

To characterize components of the recorded field potential responses, intracellular voltage recordings were obtained using Nystatin-perforation patch recording. For details of the methods, see Matsushima and Aoki (1995). Briefly, patch glass pipettes (tip size: 1–2 μm) were filled with a solution containing (in mM: K-gluconate, 123; KCl, 18; NaCl, 9; MgCl_2_, 1; EGTA, 0.2; HEPES, 10; pH adjusted to 7.3 with KOH) with a DC-resistance of 3–6 MΩ. Nystatin (Sigma-Aldrich) was added to the solution at 100 μg/ml just before recording. To load chloride (Cl^−^) intracellularly, K gluconate was substituted by equimolar KCl, 141 mM. Membrane potential was recorded with a single-electrode voltage-clamp amplifier (CEZ-3100, Nihon Kohden Co., with bridge balance mode or single electrode current clamp mode, sampling clock rate = 30 kHz, high-cut filter = 2 kHz) and stored in a computer. Data were discarded when resting membrane potential was above −50 mV or stable responses lasted for less than 30 min.

### 2.4 Histological Verification of Stimulating and Recording Sites

After recording, slices were fixated in 4% paraformaldehyde in phosphate buffer (0.1 M PB, pH = 7.2) for ca. 30 min at room temperature, embedded in gelatin and post-fixed in the same fixative for 1 day at 4°C. Sections, 50 μm thick, were cut from the slices using a microslicer (DTK-3000, Dosaka EM Co.), and then mounted on slide glasses, air dried, and stained with cresyl violet. Data were discarded when holes made by the recording electrodes were found outside of the mesopallium. The recording sites were superimposed on a representative intact section after adjusting the location of the ventricle and the boundary between the mesopallium and the nidopallium. Histological judgements were based on the chick brain atlas by Kuenzel and Masson (1988) and terminology followed nomenclature reform (Reiner et al., 2004).

## 3 Results

### 3.1 Local Synaptic Transmission and Network Dynamics of the Pallial Neural Network

#### 3.1.1 Excitatory and Inhibitory Synaptic Transmission in the IMM

**FIGURE 1:**
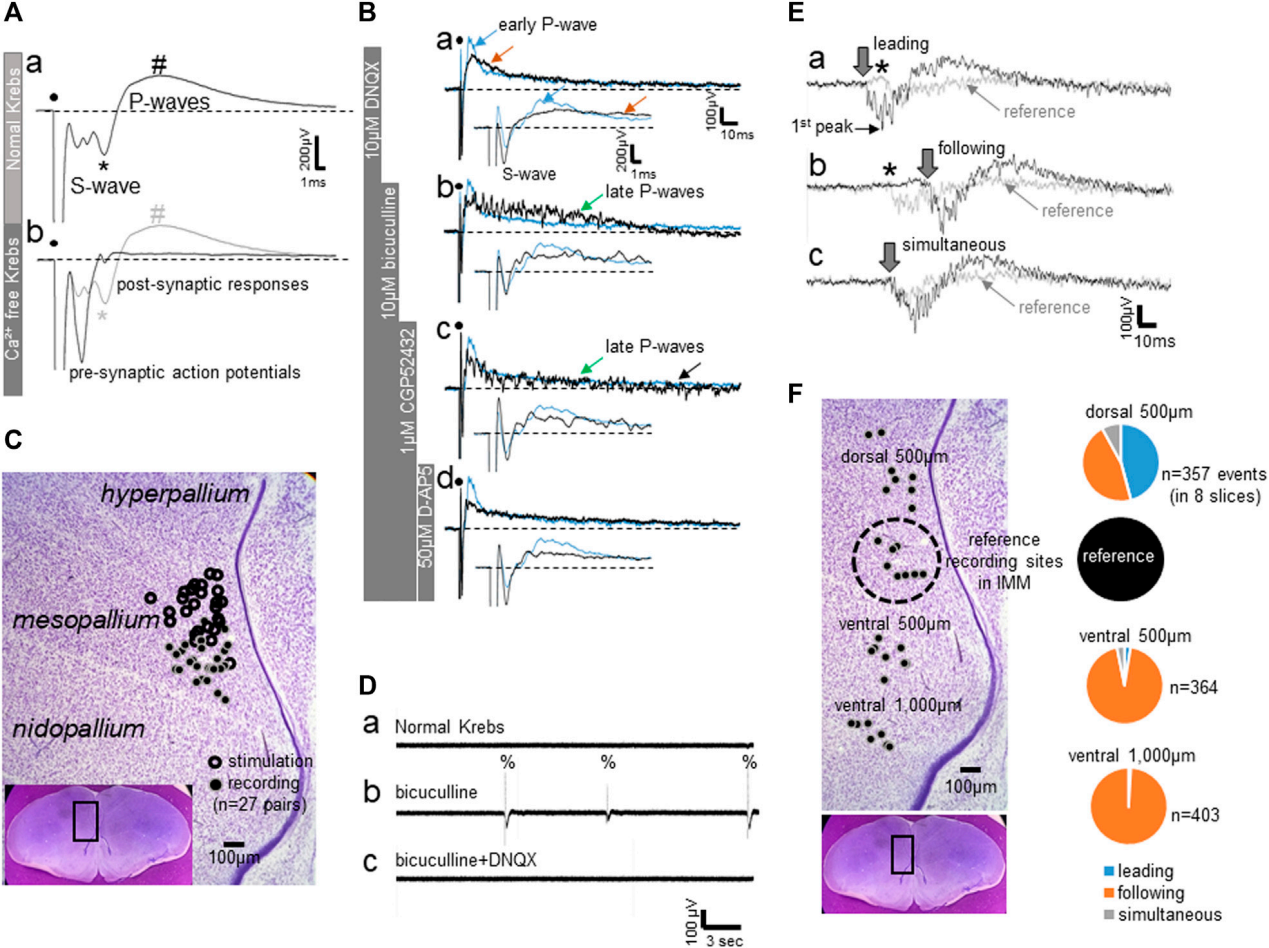
**(A–C)** Field potential responses to local electrical stimulation of the IMM. **(A)** Averaged field potentials recorded in normal Krebs and in Ca^2+^-free Krebs; ∼30 successive responses at 10 s intervals were averaged. Superimposed traces reveal a pre-synaptic component (S-wave) and post-synaptic responses (P-waves). **(B)** Bath-applied blockers dissociated components of the P-waves; single traces (not averaged). The trace shown in blue indicates the control recorded in normal Krebs. DNQX-sensitive fast excitatory transmission is represented by the early rising phase of the P-wave, whereas the late component (late P-waves) is NMDA-receptor-mediated (D-AP5-sensitive) slow excitation that was unmasked by GABA-A and–B blockers (bicuculine and CGP52432, respectively). **(C)** Sites of electrical stimulation and field potential recording plotted on a representative photomicrograph of a frontal section (see inlet). **(D–F)** Field potential recording of spontaneous epileptiform activities induced by bath-applied bicuculline. **(D)** Spontaneous epileptiform discharges (%) with bicuculline, which disappeared after DNQX was added to the bath. **(E)** Epileptiform activities simultaneously recorded from two sites, one from a point in the IMM (shown by grey traces) and another from a point 500 μm dorsal to the reference site (shown by black traces). Three examples are shown where the dorsal activity led **(Ea)**, followed **(Eb)** or occurred simultaneously **(Ec)**. Downward arrows indicate onset of the negative component, and asterisks denote the initial positive component recorded in the IMM, or the reference point. **(F)** Proportion of the leading (blue), following (orange) and simultaneous (grey) activities. Recording sites in eight slices are plotted on the same photomicrograph as C; number of events (*n*) indicates the total events obtained from these eight slices.

As reported previously (Matsushima and Aoki 1995), field potential responses to local electrical stimulation are composed of an initial negative spike-like component (S-waves) followed by slower positive waves (P-waves); Figure 1Aa shows an averaged trace (∼30 successive responses at 10 s intervals). Histological examination confirmed that stimulation electrodes were located at ca. 500 μm dorsal to the recording sites in the IMM (Figure 1C). The S-wave contained pre-synaptic action potentials that were unmasked in Ca^2+^-free Krebs solution (Figure 1Ab), whereas the later component of the S-wave (indicated by *) was of post-synaptic origin because this and the following P-waves (indicated by #) were almost completely diminished in Ca^2+^-free conditions. In other words, the post-synaptic response was composed of the initial negative (*) and the late positive wave (#) components. Subsequent successive addition of blockers to the bath (DNQX for AMPA receptor blockade, Figure 1Bb; bicuculline for GABA-A receptor blockade, Bb; CGP52432 for GABA-B receptor blockade, 1Bc; D-AP5 for NMDA receptor blockade, 1Bd) confirmed that the P-waves represents a compound of early excitatory (glutamatergic) and late inhibitory (GABA) transmission; all traces in Ba-d are non-averaged single traces. The blue trace represents a typical single response trace in normal Krebs; inlet traces show the time expansion. DNQX blocked the initial P-waves component (blue arrow in Figure 1Ba) and the late component (brown arrow). The GABA-A blocker unmasked a lasting barrage of unsynchronized spikes (green arrow in Figure 1Bb, late P-waves). Blockade of GABA-B transmission augmented the spiking barrages (black arrow in Bc), which were abolished by D-AP5 (Bd) leaving the pre-synaptic action potentials as in Figure 1Ab.

#### 3.1.2 Epileptiform Excitatory Bursts After GABA-A Blockade

In normal Krebs, without electrical stimulation, field potential recording did not show any spontaneous activities. Bath-applied bicuculline induced DNQX-sensitive epileptiform bursts (indicated by %) that emerged spontaneously at varying intervals of ca. 5–15 s (Figure 1Db). The activities were not localized but spread ventrally from the dorsomedial pallium. Field potentials were simultaneously recorded dorso-ventrally in medial forebrain regions, with the reference recording site set in the IMM (Figure 1F); experiments were repeated in eight slices. The intermediate medial hyperpallium apicale (IMHA) was 500 μm dorsal to the IMM, and the medial nidopallium (MN) was 500–1,000 μm ventral. Figure 1E shows three occasions, in which simultaneous IMHA (thick line traces, onset indicated by downward arrow) and IMM (thin line reference traces, onset indicated by asterisk) recordings were superimposed. The IMHA bursts led (Figure 1Ea) or followed (Eb) the IMM bursts in almost equal frequency, indicating that the bursts could emerge from epileptic focal spots scattered around IMHA-IMM regions. However, bursts in the MN almost always followed the IMM bursts. Within the frontal plane of the pallial network, IMHA-IMM could be the most excitable hotspot, from which bursts spread ventrally to the MN.

As described below, we then examined the effects of exogenously applied T_3_ on local synaptic transmissions and epileptiform bursts in the IMM.

### 3.2 Acute Neurophysiological Effects of T_3_

#### 3.2.1 T_3_ Enhanced the Bicuculline-Sensitive Synaptic Transmissions, Field Potential Analysis

**FIGURE 2:**
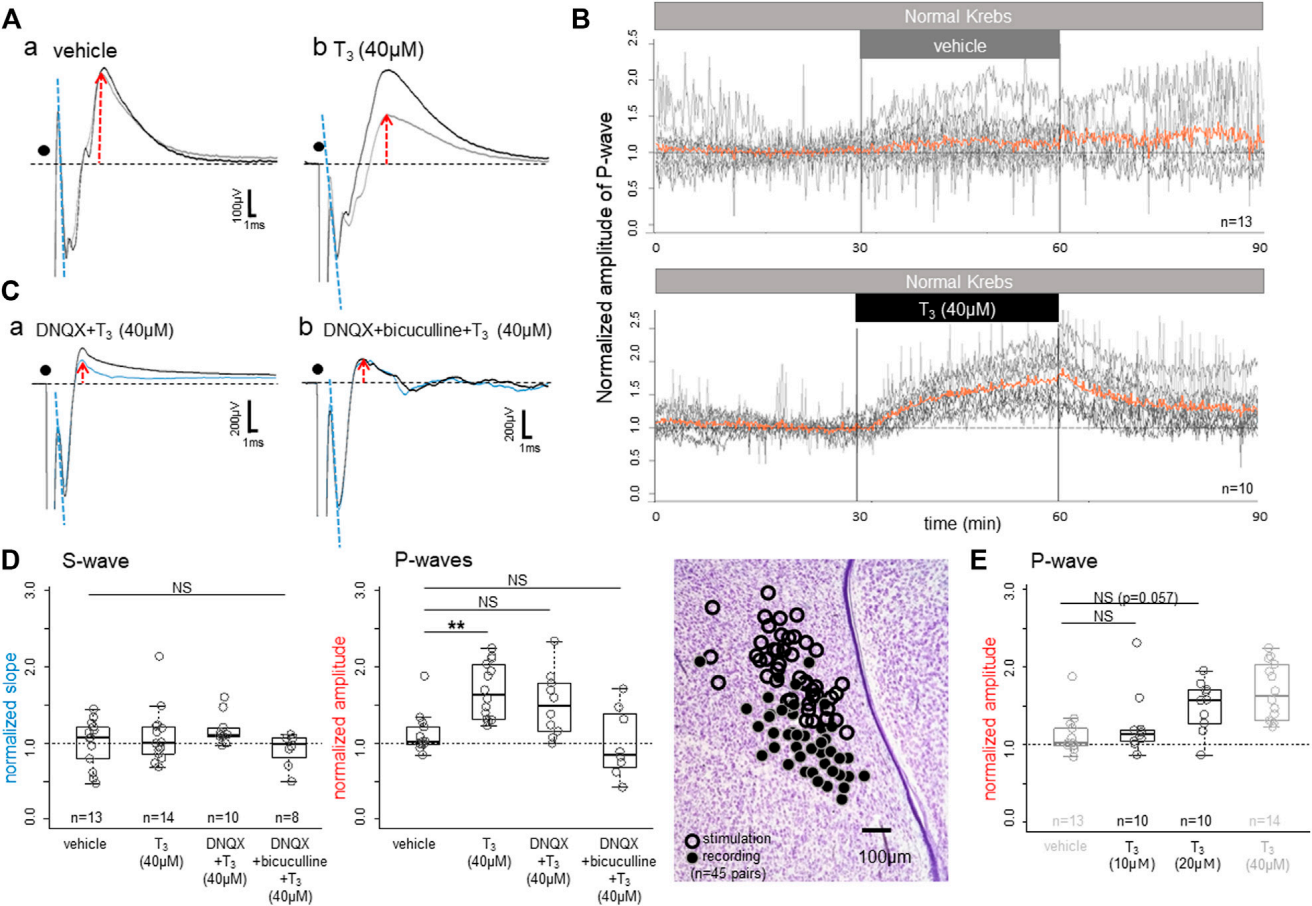
Effects of bath-applied T_3_ on the field potential responses to local electrical stimulation in the IMM. **(A)** Superimposed averaged traces (∼30 successive responses at 10 s intervals in **(A,C)** before (thin grey traces) and after (thick black traces) application of vehicle **(Aa)** and T_3_ (40 μM; **(Ab))**. **(B)** Time course of the T_3_ effect. Superimposed plots are shown for the vehicle control (top) and T_3_ (bottom); orange lines indicate the population average. **(C)** T_3_ was bath applied after the slice had been treated with DNQX **(Ca)** or DNQX + bicuculline **(Cb)**: the thin blue traces denote the pre-T_3_ responses. **(D)** T_3_ effects on the normalized slope of the S-wave (left; initial negative slope) and P-wave amplitudes (middle and right; peak amplitude). Note that the T_3_ effect survived even in DNQX, but not in DNQX + bicuculline (middle). Sites of stimulation and recording (*n* = 45 slices) are plotted. **(E)** Dose dependence of the T_3_ effects on the early P-wave; the vehicle control and the T_3_ (40 μM) data are duplicated (shown in grey) for comparison. Normalization of the slope/amplitude was done against those control records during the last 5 min (i.e., 30 trials) of the control condition before T_3_ was applied. NS denotes not significant. Asterisks denote the level of significance as; * (*p* < 0.05), ** (*p* < 0.01) and *** (*p* < 0.001).

Bath-applied T_3_ gradually enhanced the peak amplitude of the P-wave (Figure 2A); averaged traces (30 successive records at 10-s intervals) obtained from one control slice with vehicle (Aa) and another slice with T_3_ (40 μM) (Ab) are compared. Responses recorded at 25–30 min after the onset of drug application (black traces) were superimposed on those during a 5-min period just before the application (grey traces with red upward arrows). Effects were not found on the onset slope of the initial S-wave (indicated by dashed blue lines), indicating that the neuronal excitability to the applied electrical stimulation remained unchanged.

Figure 2B shows the time course of changes in the vehicle control (upper) and the T_3_ experimental (lower) slices. Recording was composed of pre-application (0–30 min), application (30–60 min) and post-application wash-out (60–90 min). Peak amplitude of the P-wave was plotted every 10 s after normalization against the responses during the last 5 min of the pre-application. Red traces indicate the population average of the superimposed recordings (13 slices for vehicle and 10 slices for T_3_). The P-wave amplitude started to rise as soon as T_3_ was introduced to the bath and rose continuously during the application period. After T_3_ was removed from the bath, the P-wave dropped immediately, but the amplitude stayed higher than 1.0 at 90 min, at 30 min after washout.

The T_3_ effects survived after AMPA receptor-mediated excitatory transmission was blocked by DNQX (Figure 2Ca) but disappeared when GABA-A receptor was also blocked (Cb). Also note that DNQX strongly suppressed the P-wave; when compared with the control traces (gray traces in Aa,b), P-wave was much smaller in DNQX (blue traces in Ca). Population data are shown in Figure 2D for the normalized slope of the S-wave (the initial drop phase; dashed blue lines) and the peak amplitude of the early P-wave (normalized against the pre-T_3_ perfusion; red arrows). One-way ANOVA revealed no significant difference in the S-wave (*p* = 0.28), but the differences were significant in the early P-wave (*p* = 0.0016); further post-hoc multiple comparisons were made by Tukey’s method and revealed a significant difference between vehicle and T_3_ (40 μM). The dose response relationship of T_3_ effects was examined in an additional 20 slice preparations as shown in Figure 2E. Comparisons with the duplicated data of Figure 2D revealed a significant effect of dose (ANOVA, *p* < 0.001), although post-hoc multiple comparisons showed only a suggestive level of difference (*p* = 0.057) for T_3_ (20 μM) over vehicle.

These findings indicated that T_3_ enhances GABA-A-ergic inhibitory synaptic transmission without changes in the pre-synaptic action potentials, namely the neuronal excitability. The early P-waves are considered to be composed of GABA-A-ergic synaptic inhibition. At this post-hatch stage, GABA-A receptor mediated synaptic current is hyperpolarizing by Cl^−^ influx (see below and Figure 5Bb for the reversal potential of the Cl^−^ ion), giving rise to positive waves when recorded extracellularly as field potential response. However, these results do not reject the possibility that the AMPA receptor-mediated fast synaptic excitation is also modulated by T_3_.

#### 3.2.2 T_3_ Failed to Facilitate Synaptic Potentiation by Low-Frequency Tetanic Stimulation, Field Potential Analysis

**FIGURE 3:**
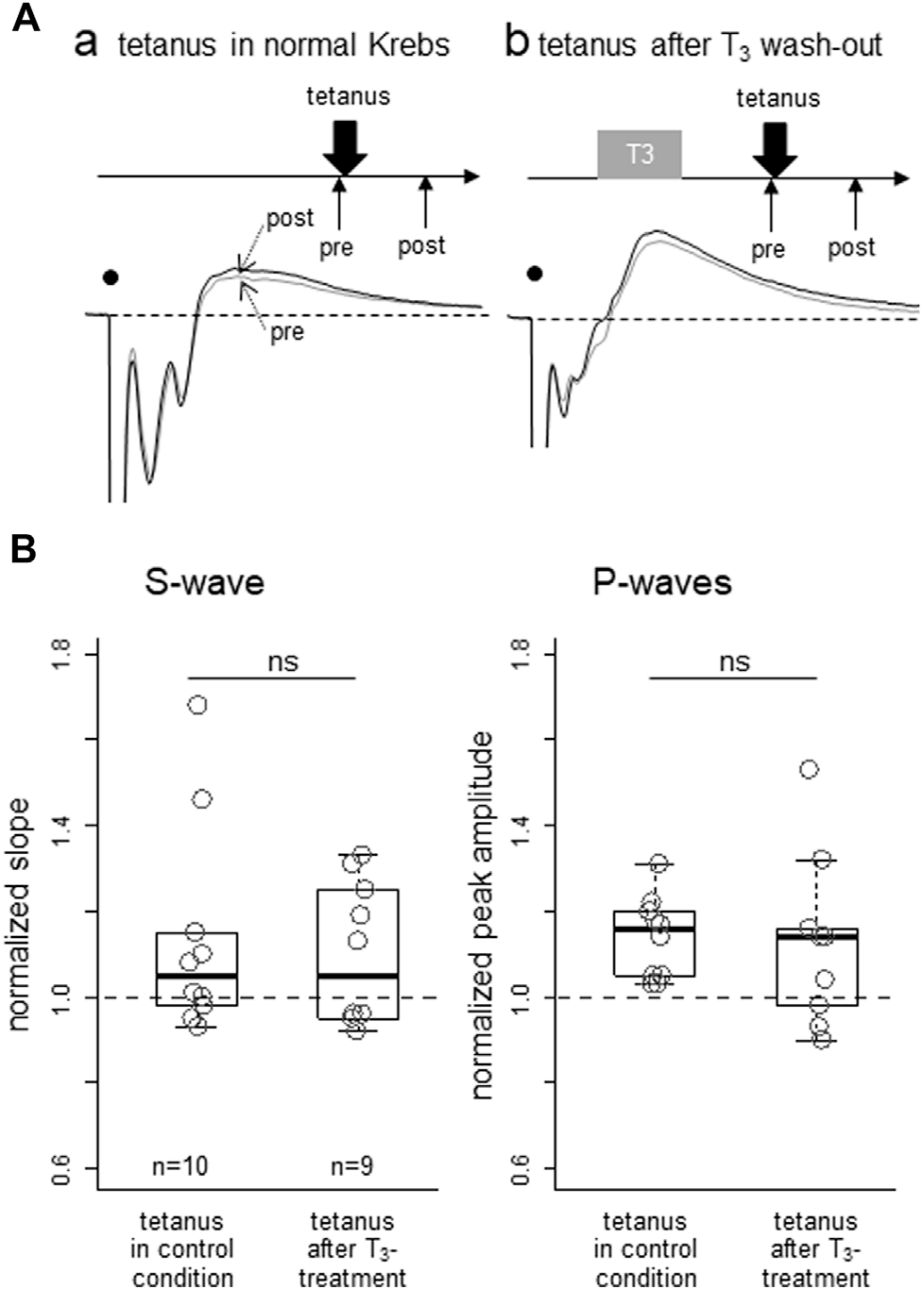
Lack of T_3_ effect on lasting potentiation of synaptic responses after low-frequency tetanic stimulation applied locally in IMM. Effect of T_3_ pre-treatment on the subsequent induction of potentiation was examined. **(A)** Two examples of pre- (thin) and post-tetanus (thick) traces, one with T_3_ pretreatment **(Aa)** and one without **(Ab)**; ∼30 successive responses at 10 s intervals **(B)** Normalized initial negative slope of the S1-wave (left) and normalized peak amplitude of the early P-wave were compared between two groups, but no significant difference was apparent.

To examine if T_3_ could also modulate activity-dependent synaptic plasticity, effects of low frequency tetanic stimulation were compared between two groups of slice preparations, one with T_3_ bath application for 30min (with an additional interval of 30min before tetanus) and another without T_3_ (control). In both groups, slice preparations were maintained in the recording chamber with test stimulation applied at 10-s intervals. As shown in Figure 3A (averaged traces for 5 min from two examples) and Figure 3B (population data), pre-treatment by T_3_ yielded no significant differences; S-wave (*p* = 0.71; Mann-Whitney’s *U*-test) and early P-wave (*p* = 0.49), respectively. As the slice preparations were obtained from naïve inexperienced chicks (see 2.1. in Methods), we assume that non-specific saturation of synaptic plasticity did not occur as reported by Bradley et al. (1999).

#### 3.2.3 T_3_ Suppressed the Frequency of the Epileptiform Bursts Induced by Bicuculline, Field Potential Analysis

**FIGURE 4:**
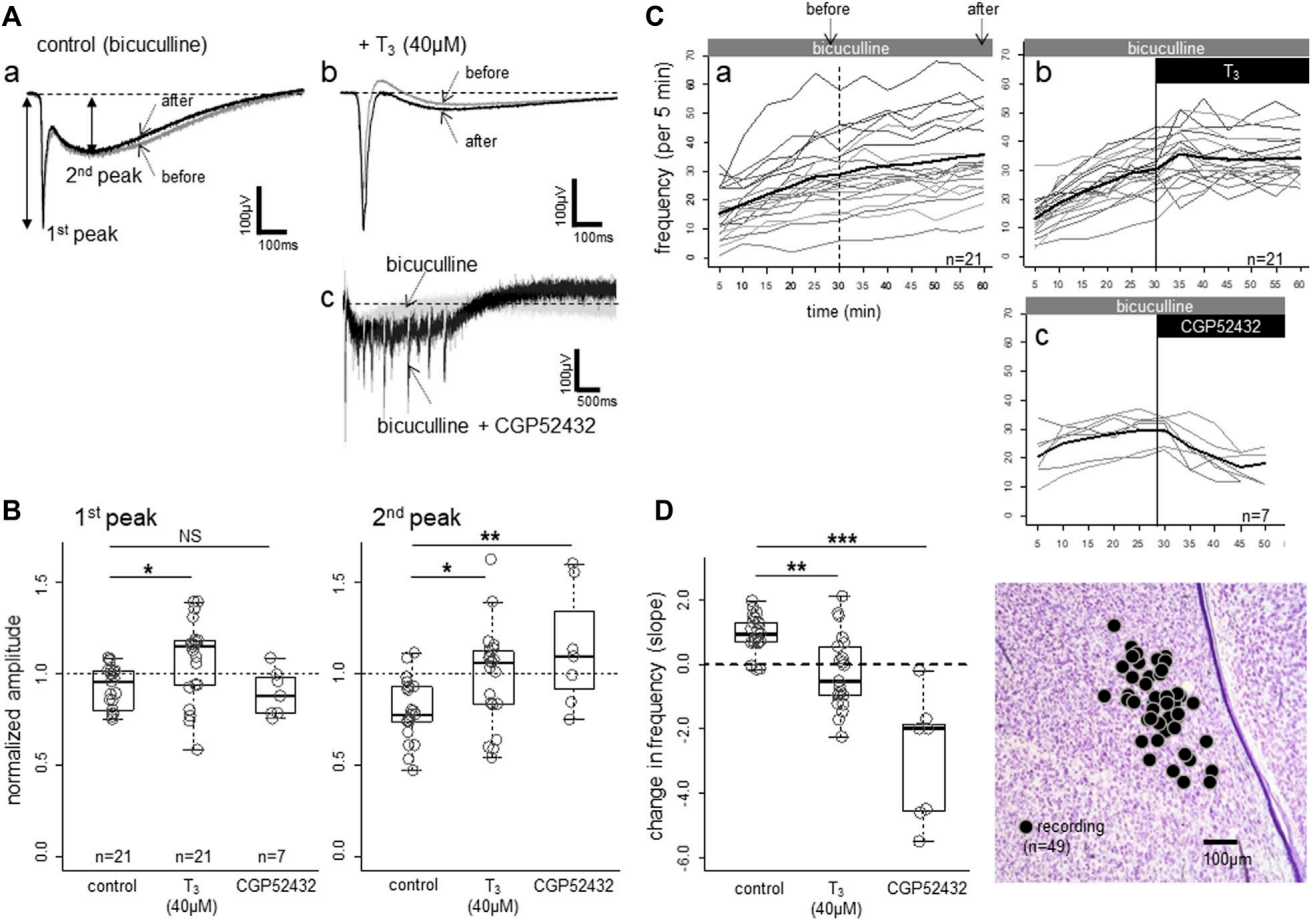
Effects of bath-applied T_3_ (40 μM) on bicuculline-induced spontaneous epileptiform activities. **(A,B)** T_3_ effect on the waveform. The activities were composed of a sharp negative component (1st peak) followed by a slower negative component (2nd peak), both of which were larger in T_3_. CGP52432 (GABA-B blocker) in the bath caused a lasting barrage of sharp negative components. **(C,D)** T_3_ effects on frequency (number of events per 5-min bin; thin lines indicate individual slices and thick line the average. Note a gradual increase in the control slices. Change in frequency of events (per 5 min bin) was fitted to linear lines for the data of each slice, and the estimated slope of the change was plotted and compared. Sites of recording (*n* = 49) plotted on the same photomicrograph as Figures 1, 2. Asterisks denote the level of significance as; * (*p* < 0.05), ** (*p* < 0.01) and *** (*p* < 0.001).

The spontaneous epileptic bursts induced by bicuculline are composed of two negative components. The first peak (Figure 4Aa and Figure 4b; also see Figure 1E) represents the highly synchronized compound action potentials due to fast excitatory transmission, and the second peak coincides with the lasting strong depolarization of the NMDA receptor mediated plateau potential (Yanagihara et al., 1998). The bursts repeated for several hours, and the amplitude (both 1st and 2nd peaks) gradually became smaller (Figure 4Aa); superimposed traces recorded at 30 min (grey trace) and 60 min (black trace). The gradual decrease in amplitude was accompanied by gradual increase in frequency (number of bursts in 5 min, Figure 4Ca). Further blockade of GABA-B-ergic transmission by CGP52432 (Figure 4Ac) strongly enhanced the 2nd peak, on which barrages of epileptiform bursts were superimposed for several seconds. These barrages were suppressed by D-AP5 (data not shown), indicating that GABA-B transmission potently masked the NMDA receptor mediated long-lasting excitation of the network. However, CGP52432 failed to change the first component (1st peak in Figure 4B), which is the fast compound action potentials.

T_3_ increased the amplitude and decreased the frequency of the epileptic bursts. Population data (Figure 4B) revealed that both components were enhanced by T_3_; one-way ANOVA revealed significant differences among the three groups (*p* = 0.015 for the 1st peak, *p* = 0.038 for the 2nd peak), with both peaks showing significant pair-wise differences between control and T_3_ (post-hoc Tukey’s multiple comparisons). In the control condition in which only bicuculline was applied, the frequency gradually increased (Figure 4Ca). When T_3_ (40 μM) was added, the frequency increase was arrested (Cb), and the slope of the frequency change during the latter half of the recording session (30 min) was significantly lower (Figure 4D) (*p* = 0.003; Tukey’s multiple comparison). CGP52432 caused a much stronger suppression (*p* < 0.001) compared with the control and T_3_.

These T_3_ effects on epileptiform bursts can be accounted for either by 1) enhanced NMDA receptor mediated depolarization or 2) reduced GABA-B receptor mediated inhibition. Furthermore, T_3_ could modulate the resting membrane potentials, spiking threshold and other factors involved in spike generation, rather than synaptic transmissions. We have therefore examined T_3_ effects by intracellular recordings from IMM neurons.

#### 3.2.4 T_3_ Suppressed the NMDA Plateau Potential and Enhanced GABA-A Responses, Intracellular Whole-Cell Recording Analysis

**FIGURE 5:**
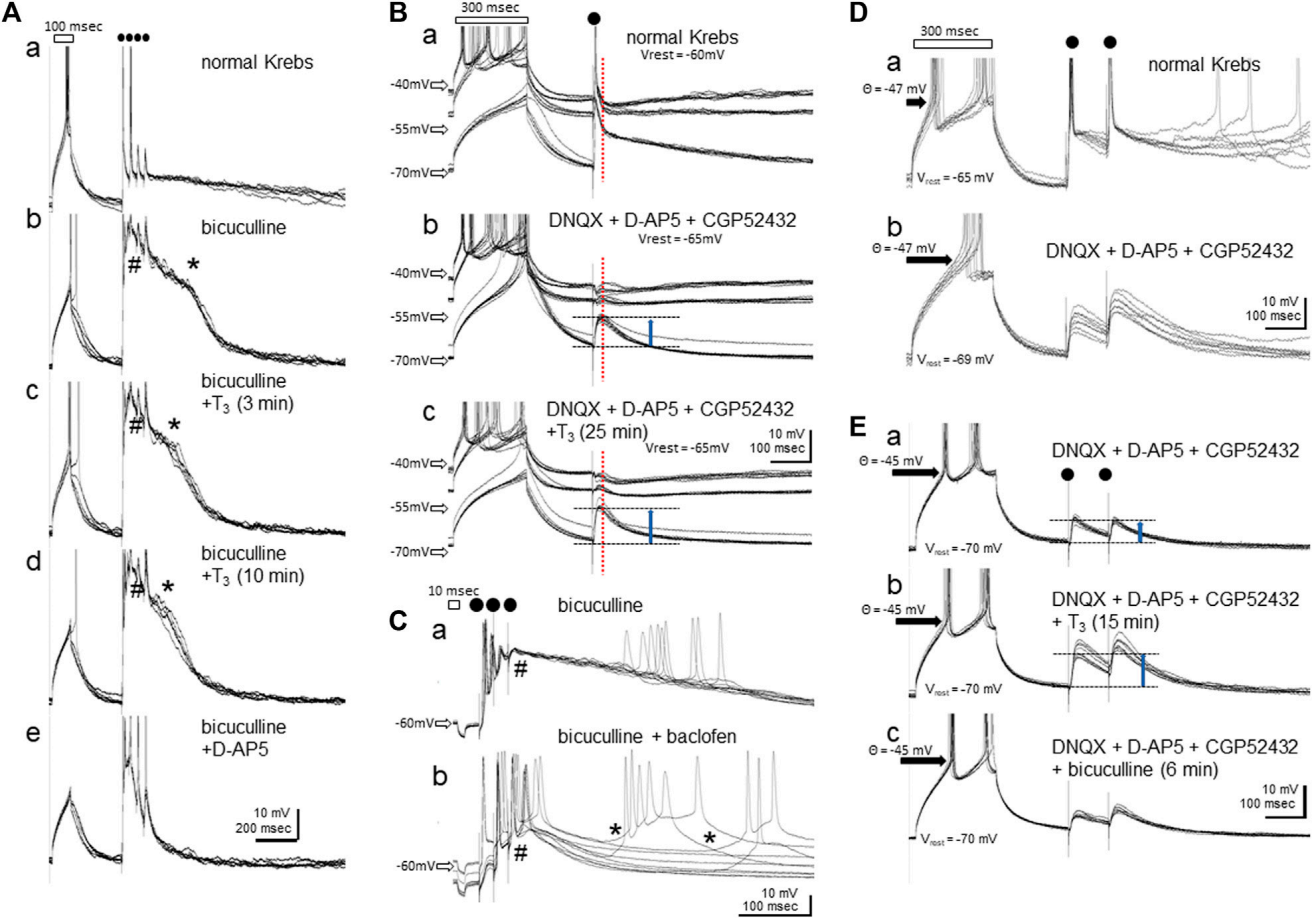
Effects of bath-applied T_3_ (40 μM) on the membrane potentials in IMM neurons. **(A–C)** show recordings using normal Cl^−^ pipette solution. **(D,E)** show recordings with high-Cl^-^ electrode and GABA-A responses revealed as depolarization. **(Aa-e)** T_3_ suppressed the NMDA-plateau. Initial depolarizing current injection (indicated by a rectangle, 100 msec) was followed by four repetitive local electric shocks (dots); # indicates the initial onset phase of the NMDA plateau potential by bicuculline, and * the shoulder (onset of the decaying phase) of it. Six consecutive traces recorded at 10 s intervals are shown for each. **(Ba-c)** Responses recorded at three steps of tonic current injection through the recording electrode. Vertical dashed red line indicates the peak GABA-A response that is reversed at ca. −50 mV. Vrest means the resting membrane potential without current injection. **(Ca-b)** Baclofen induced sporadic NMDA-plateau depolarization (*). **(Da-b)** Blockade of AMPA, NMDA and GABA-B receptors unmasked GABA-A receptor mediated depolarization. **(Ea-c)** Further addition of T_3_ enhanced the GABA-A depolarization (upward blue arrows). No effects were found in the resting membrane potential (V_rest_ = −70 mV), membrane resistance, or threshold for spike generation (θ = −45 mV).

NMDA receptor activation produces a bistabile property in the membrane potential by negative resistance because of depolarization-induced release of the Mg^2+^ blockade. When disinhibited by bicuculline, local electrical stimulation reliably elicited a plateau potential for a considerable period without any additional synaptic drive. Figure 5A shows an example recorded in single-electrode current clamp mode using a normal (low Cl^−^ concentration) pipette solution. After a depolarizing current injection through the recording electrode (duration = 100 msec, indicated by a rectangle above the traces), four repetitive electric shocks were applied at 40 msec intervals (dots); six consecutive responses recorded at 10 s intervals were superimposed. The depolarizing current intensity was adjusted so that one action potential was induced at the peak of the depolarization. In control conditions (normal Krebs in the bath), each shock generated quickly decaying excitatory post-synaptic potentials (EPSPs) with action potentials on the peak of the first and/or the second shocks. When bicuculline was added (Ab), a depolarizing plateau (indicated by * at the shoulder) appeared and lasted for ca. 400 msec after the electric shocks; notice that the depolarization muted the neuron and action potentials failed to occur during the plateau. Bath applied T_3_ (40 μM, Ac and Ad) gradually reduced the duration of the plateau (3 and 10 min after T_3_ reached the chamber) without suppressing the initial component (indicated by #); fast EPSPs were thus supposed to be insensitive to T_3_. D-AP5 completely suppressed the plateau (Ae), confirming its NMDA-receptor origin. Notice also that the resting membrane potential, the depolarization by the current injection, and the threshold membrane potential for generating spikes remained unchanged.

The NMDA-plateau potential was induced by bicuculline application in all 15 neurons recorded from eight slice preparations, with resting membrane potentials ranging from -50 to -67 mV (mean ± s. e.m. = -59.5 ± 1.6 mV) and stable recording maintained for 40–84 min. T_3_ effects (40 μM, >15 min bath application) were successfully examined in 11 neurons, and the NMDA plateau was suppressed by T_3_ in 10 out of these 11 neurons; no changes were detected in the remaining one neuron. Vehicle control was tested in other four neurons, and none of them showed changes in the plateau.

The GABA-A receptor-mediated inhibitory synaptic response was examined after glutamatergic (AMPA- and NMDA-R mediated) and GABA-B ergic transmission was blocked (Figure 5B, bridge balance mode). In normal Krebs (Ba), tonic current injections at three levels (two depolarizing and one hyperpolarizing) set the membrane potential at ca. −45 mV, −50 mV and −70 mV. Single shocks of local electrical stimulation elicited fast EPSPs, and single action potentials followed by a hyperpolarization at −45 mV (indicated by vertical dashed red line). The hyperpolarization phase turned to depolarization if held at −70 mV, indicating the reversal of the GABA-A response at approximately −50 mV. After glutamatergic and GABA-B-ergic transmission were blocked by DNQX, D-AP5 and CGP52432 (Bb), the resting membrane potential was hyperpolarized by 5 mV. Synaptic responses were examined by adjusting the tonic current injection levels. GABA-A ergic depolarization was clear at −70 mV (upward blue arrow). When T_3_ was added (40 μM, Bc), the depolarizing GABA-A response increased without changes in the reversal potential, resting potential, or threshold of spike generation. The bicuculline-induced lasting depolarization was further augmented by baclofen (20 μM) (Figure 5Ca,b); note that the synaptically driven initial depolarization (indicated by #) was suppressed, thus allowing slow depolarization of varying latency and amplitude (indicated by *). The GABA-B receptor activation paradoxically facilitated the spontaneous bursting caused by NMDA receptor activation. Actually, the delayed depolarization was not accompanied by synaptic drives if measured in voltage clamp mode and disappeared with the addition of D-AP5 (data not shown). Most probably, the hyperpolarizing effect of baclofen via GABA-B receptor activation set the membrane potential within an appropriate range for the NMDA-induced plateau to spontaneously arise.

The GABA-A responses were examined in nine neurons from four slices, with resting membrane potentials ranging from −55 to −69 mV (mean ± s. e.m. = -63.9 ± 1.5 mV), and T_3_ was tested in eight neurons and vehicle in 1 neuron. Of these eight neurons, enhancement by T_3_ occurred in six, suppression in one and no detectable effect was found in another. The effect of baclofen on the NMDA plateau was reproduced in all of the other three neurons tested. Because the reversal potential of the GABA-A response was close to the resting potential (usually ∼5mV above than the resting level), a small accidental change in the resting level had a big influence on the GABA-A response amplitude. We therefore re-examined the effects of T_3_ using high-Cl^-^ pipette solution.

Increase in the GABA-A response by T_3_ was confirmed (Figures 5D,E). At the resting membrane potential, the GABA-A response quickly turned depolarizing after poking the neuron, and reached equilibrium within ∼10 min. In normal Krebs, two pulse electric shocks (100 msec interval) induced initial spiking followed by lasting depolarization (Da). The depolarizing response remained after DNQX, D-AP5 and CGP52432 were added (Db). The unclamped resting membrane potential was slightly hyperpolarized (from −65 to −69 mV) but the spiking threshold potential (−47mV) remained unchanged. In another neuron (Ea), T_3_ enhanced the depolarizing response (Eb). The depolarization was suppressed by bicuculline (Ec) although not completely. We therefore argue that GABA-A receptor mediated responses was enhanced by T_3_. T_3_ application caused no changes in the spiking threshold (*θ* = −45 mV) in response to a rectangular depolarizing current injection through the recording electrode.

The reversed GABA-A potential was reliably recorded from six neurons from five slice preparations, with resting membrane potentials ranging from −59 to −77 mV (mean ± s. e.m. = −68.7 ± 3.1 mV), and T_3_ effects (>15 min of bath application) successfully examined in all six neurons. Enhancement by T_3_ occurred in five out of these six neurons, whereas suppression occurred in the other neuron.

The possibility that T_3_ enhances the NMDA-receptor mediated response (see Section 3.2.3) should therefore be rejected, and an alternative hypothesis must be considered such that GABA-B mediated inhibition was suppressed. Taken together, T_3_ could have opposite effects on the GABA-mediated responses, namely, enhancement of the fast GABA-A-mediated inhibition and suppression of the slow GABA-B-mediated inhibition of the IMM network.

## 4 Discussions

### 4.1 Thyroid Hormone, the Issue of the Effective Dose

Our results showed that bath application of T_3_ at 20–40 μM reliably modified synaptic transmission, namely it enhanced GABA-A and suppressed NMDA responses. In our previous behavioral pharmacology study (Yamaguchi et al., 2012), we reported that single injection of a small amount of T_3_ (10 μM x 100 μl, intravenous injection) effectively re-opened the sensitive period. The low dose effects of thyroid hormones have been confirmed by Batista et al. (2018) for the T_4_ effect on imprinting, Miura et al. (2018) for the T_3_ effect on biological motion preference, and Lorenzi et al. (2021) for the T_3_ effect on animacy preference. We must consider two possible explanations for the discrepancy. Systemically injected T_3_ could be diluted to sub-micromolar level in the circulation and brain tissue, and the high dose effects found in this study are not supposed to be physiological. Alternatively, the thyroid hormone transporter system in the dorsal telencephalon could accumulate T_3_ in the peri-vascular neural tissue, and the concentration could reach a supra-micromolar level in the peri-neuronal space.

In favor of the latter possibility, localized infusion of the monocarboxylate transporter inhibitor (bromsulphthalein) to IMM effectively nullified the effects of systemically injected T_3_ (Yamaguchi et al., 2012). The transport and accumulation mechanism may not function in the slice preparations and the actual effective dose may be supra-micromolar. Accordingly, in rat primary hippocampal neuron culture (Losi et al., 2008), T_3_ inhibited the NMDA current at IC_50_ ∼ 15 μM. Although the opposite T_3_ action to that in our present study, these authors reported that T_3_ inhibited GABA current at IC_50_ ∼ 13 μM (Puia and Losi, 2011). When systemically applied *in vivo*, however, repeated i. p. injection of levothyroxine was effective at a low dose of 20 μg (∼26 nano mole)/kg/day in rescuing the hypothyroidism-induced cognitive impairment (Alzoubi et al., 2009). This dosage was several magnitudes lower than that applied to cultured neurons. Changes in any of the following processes could contribute to the effectiveness of thyroid hormone action: synthesis and release of thyroxine (T_4_) by the thyroid gland, conversion of T_4_ to T_3_ at the target organ (vascular endothelial cells) and transportation to the peri-neuronal space.

It may also be interesting to examine the T_4_ action *in vitro*, even though T_3_ is supposed to be critical player as argued above. Because we do not yet fully understand the mechanism of the T_3_ action, several interesting possibilities arise. T_4_ may act as T_3_ at similar dose *in vitro* but has no functional significance because the brain content of T_4_ is nearly at the detectable level (Yamaguchi et al., 2012, Figure 3A). Alternatively, we may assume that T_4_ acts differently, so that the T_3_ action is modulated by T_4_ at the extremely low concentration. Further studies are needed to address these possibilities, but only after we are quite sure about the T_3_ action.

### 4.2 Opposing Roles of GABA-A and B Receptors

Our recent qPCR analysis revealed that GABA-A receptor expression increases from day1–5, whereas GABA-B expression gradually decreases during this early post-hatching period (Aoki et al., 2018). Therefore, the ratio of GABA-A to -B receptor (referred to as the A/B ratio below) gradually increases. We have also found that direct infusion of CGP-52432 (GABA-B antagonist, thus increasing the A/B ratio) into the IMM suppressed imprinting on day 1. However, infusion of baclofen (GABA-B agonist) or bicuculline (GABA-A antagonist) decreased the A/B ratio and made 4-day old chicks imprintable as on day 1. Based on these effects, we hypothesized that a low A/B ratio could be one of the neurophysiological phenotypes of the sensitive period of imprinting. Importantly, the GABA-A and B receptors could have opposing roles in the regulation of the sensitive period. One possible explanation is that activation of GABA-B receptors on pre-synaptic terminals leads to suppression of GABA release from inhibitory interneurons (Gassmann and Bettler, 2012; Aoki et al., 2018), thus excitability of the IMM network is enhanced through GABA-B-mediated suppression of the Ca^2+^ influx into presynaptic terminals, which is responsible for GABA release (Deisz and Prince, 1989).

The A/B ratio increase during the days 1–5 post-hatching means a monotonic decrease in network excitability. The present finding that the GABA-B agonist, baclofen, induced the NMDA-plateau (Figure 5Cb) supports this possibility. An appropriate decreased in the A/B ratio seems to be necessary for spontaneous NMDA bursting to occur in the IMM network. GABA-A blockade alone (Ca) strongly depolarizes neurons, thereby muting action potentials, whereas additional activation of GABA-B lowers the membrane potential to around the NMDA plateau, so that short barrages of synaptic inputs can cause lasting spiking responses after transient synaptic drives. Alternatively, as GABA also acts on somatic receptors extra-synaptically via volume transmission (Matsushima et al., 1993; Tegnér et al., 1993), activation of GABA-B receptors and the associated G-proteins may have directly enhanced the NMDA-plateau of the recorded neurons. Further analyses are needed to fully understand how GABA controls the dynamics of the IMM network.

Contrary to our initial expectation, T_3_ was a potent enhancer of the GABA-A response (Figures 5B,E), thereby increasing the A/B ratio. As T_3_ partially mimicked the effect of CGP52432 on the bicuculline-induced spontaneous bursts (2^nd^ peak amplitude and frequency; Figure 4Ab and Figure 4B), T_3_ may suppress GABA-B so that the A/B ratio is further decreased. Blockade of GABA-B receptors on pre-synaptic terminals of inhibitory interneurons would disinhibit GABA release, so that the post-synaptic GABA-A response would be enhanced. However, the neuronal cascades through which T_3_ facilitates imprinting remain to be clarified.

Why T_3_ target on GABA? We have yet no clear idea about the functional link between the central thyroid hormone effect and the imprinting memory formation. Beside the link discussed above, we may assume that immediate early genes (such as *c-fos*) could be linked, as imprinting is reported learning-related increase in Fos-immunoreactivity specifically in GABA neurons (Ambalavanar et al., 1999). The thyroid hormone influx to IMM might lead to the enhanced GABA release through expression of those genes selectively in the GABA-ergic inhibitory interneurons, and the GABA release mechanism is downstream to the c-fos.

### 4.3 NMDA-Plateau Potential and Activity-dependent Synaptic Potentiation

McCabe et al. (1992) reported that blockade of NMDA receptors in the dorsomedial pallium impairs imprinting, while imprinting upregulates the glutamatergic receptors in the IMM (McCabe and Horn, 1988), subsequently leading to morphological and functional changes in IMM synapses (Horn, 1998). Accordingly, DNQX-sensitive fast excitatory transmission was potentiated by a low-frequency tetanic stimulation (Matsushima and Aoki, 1995), which turned out not to be linked to the immediate early gene expression associated with memory (Yanagihara et al., 1998). Two decades later, our understanding of imprinting remains limited, mostly because we do not know what activities occur in the IMM network during imprinting *in vivo*, nor which activities are critical for imprinting-related memory formation.

Dynamic behaviors of the IMM neurons *in vitro* could give us a hint about their *in vivo* behaviors. During the low-frequency tetanic stimulation for 1 min (5 Hz × 300 pulses), GABA-A mediated synaptic inhibition quickly fades away, and is replaced by NMDA-receptor mediated slow depolarization (Matsushima and Aoki, 1995). If the bicuculline-induced bursts observed in slice preparations paralleled the activities assumed *in vivo*, facilitatory modulation of the 1st and 2nd spike by T_3_ (Figure 4Ab, Figure 4B) would underlie the initial phase of memory formation, rather than the subsequent potentiation of the post-synaptic responses (Figure 3).

In this study, we could not specify whether T_3_ action was genomic or nongenomic. At the behavioral level, T_3_ facilitated imprinting within a few hours after injection. In slices, bath applied T_3_ changed the synaptic transmission immediately (within a few min; Figure 2B), in favor of non-genomic effects. Naturally, the late phase of the T_3_ actions could be genomically caused. At present, we are unable to dissociate these two processes.

## Data Availability

The raw data supporting the conclusion of this article will be made available by the authors, without undue reservation.
